# A Comparative Study of Formic Acid, Herbal Mixture and Spirulina Powder as Antibiotic Alternatives in Broiler Diets: Effects on Growth, Carcass Traits, Blood Biochemistry and Microbial Load

**DOI:** 10.1002/vms3.70986

**Published:** 2026-05-05

**Authors:** Elwy A. Ashour, Saad A. Al‐Ardhi, Dalia A. A. Elsayed, Mahmoud Moustafa, Islam M. Youssef, Mohammad M. H. Khan, Mohamed E. Abd El‐Hack

**Affiliations:** ^1^ Poultry Department, Faculty of Agriculture Zagazig University Zagazig Egypt; ^2^ Department of Animal Production Agriculture College, Al‐Muthanna University Samawah Iraq; ^3^ Department of Poultry Breeding, Animal Production Research Institute Agricultural Research Center Giza Egypt; ^4^ Department of Biology, College of Science King Khalid University Abha Saudi Arabia; ^5^ Animal Production Systems Research Department Animal Production Research Institute, Agricultural Research Center Giza Egypt; ^6^ Department of Biochemistry and Chemistry Faculty of Biotechnology and Genetic Engineering, Sylhet Agricultural University Sylhet Bangladesh; ^7^ Department of Industrial Pharmacy, College of Pharmaceutical Sciences and Drug Manufacturing Misr University for Science and Technology (MUST) Giza Egypt

**Keywords:** broilers, formic acid, growth performance, herbal mixture, spirulina powder

## Abstract

**Background:**

Restrictions on antibiotic growth promoters in poultry production have driven the search for safe, natural alternatives. Organic acids, herbal additives and spirulina are promising options due to their antimicrobial, antioxidant and growth‐enhancing effects.

**Objectives:**

The purpose of this experiment was to evaluate the effects of formic acid (FAc), herbal mixture (HMX) and spirulina powder (SPI) as potential antibiotic alternatives in broiler diets, specifically examining their impact on growth, carcass characteristics, blood biochemistry and intestinal microbial count.

**Methods:**

Six replicates of ten unsexed chicks per group comprised the eight experimental groups to which 480 one‐day‐old Ross 308 broiler chicks were randomly assigned. The treatments were as follows: T1 (control); T2 (0.5 g Colistin antibiotic/kg diet); T3 (2 cm^3^ FAc/kg diet); T4 (4 cm^3^ FAc/kg diet); T5 (2 g HMX/kg diet); T6 (3 g HMX/kg diet); T7 (0.5 g SPI/kg diet); and T8 (1 g SPI/kg diet).

**Results:**

Significant differences were detected in daily body weight gain (DBWG) and live body weight (LBW), with the spirulina‐treated groups (T7 and T8) showing the highest values. Also, significant influences on feed intake (FI), feed conversion ratio (FCR) and performance index (PI), with the HMX groups showing the most favourable FCR and the highest PI. Blood biochemistry and antioxidant markers were influenced by the HMX and SPI treatments. The SPI groups showed reduced liver enzyme levels, while the HMX groups demonstrated improved protein profiles and enhanced antioxidant enzyme activity. Microbial analysis revealed that FAc and HMX treatments led to a reduction in pathogenic bacteria, with T3, T4 and T5 showing the lowest levels of *Escherichia coli* and *Salmonella*.

**Conclusion:**

These results suggest that FAc, HMX and SPI are promising dietary supplements for enhancing both the broiler chickens' growth rates and general health.

## Graphical abstract

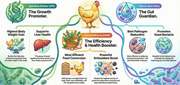

## 1 Introduction

The demand for chicken products has increased due to the significant growth in the world's population, making it necessary to supply this demand while maintaining the quality and safety of the food. Traditional antimicrobials, or antibiotics, have long been used for the treatment and prevention of bacteria‐related infections in animal production, particularly in poultry. However, antimicrobial drug resistance, resulting from the overuse and misuse of these substances, has become a major public health concern (Nakhaee and Hafez 2025; Zhu et al. 2025). Producing sufficient food to feed the global population remains one of the world's maximum pressing challenges (Sousa et al. 2024). Furthermore, the extensive use of antibiotics in livestock husbandry, combined with the growing number of animals raised in intensive farming systems, has contributed to the development and extent of antibiotic resistance, raising significant concerns for public health worldwide (Youssef, Elsherbeni, et al. 2024; Youssef, Aldhalmi, et al. 2024).

In response to this issue, natural feed additives have gained popularity in recent years as replacements for synthetic growth promoters and antibiotics in animal nutrition programs. These additives help to enhance immune and efficiency through decreasing or managing the microbial load in the guts (Youssef, Khalil, Jaber, et al. 2023, Youssef, Khalil, Shakoori, et al. 2023, Youssef, Khalil, Swelum, et al. 2023; Dosoky et al. 2024; Cufadar et al. 2024). Many herbs possess biologically effective properties that benefit animal feeding, and their use in livestock production has been practiced for centuries. The antimicrobial, immune‐fuelling, anti‐inflammatory and antioxidant abilities of herbs are well known (Abd El‐Hack et al. 2025). Herbal mixtures (HMX) were used as natural feed treatment additions and substitutes before the 20th century's introduction of antibiotic growth promoters (AGPs) (Adli et al. 2024; Abou‐Kassem et al. 2025). However, as the effectiveness of synthetic drugs declines and more contraindications to their use emerge, natural feed additives are once again gaining importance (Emmanuel et al. 2024).

Dietary acids can be classified as organic or inorganic, with organic acids (OAs) widely applied in poultry nutrition. Beyond their antimicrobial action, OAs exert important physiological effects in broilers by regulating gastrointestinal pH, suppressing pathogenic bacteria and promoting beneficial microflora, thereby improving gut health (Acharya and Barsila 2025). Due to their favourable physicochemical properties and efficient absorption, short‐chain OAs such as butyric, propionic, lactic, malic, tartaric, fumaric and acetic acids are commonly used in poultry diets (Abd El‐Ghany 2024). OAs enhance digestive enzyme secretion, improve intestinal morphology (villus height and villus‐to‐crypt ratio) and increase nutrient and mineral (Ca, P, Zn) digestibility and utilization, supporting metabolic efficiency and immune responsiveness (Zhang et al. 2025). By creating a favourable intestinal environment, OAs improve feed intake (FI), growth performance, feed efficiency and disease resistance, which has led to extensive evaluation of FAc supplementation for its physiological and growth‐promoting effects in broilers (Ebeid and Al‐Homidan 2022; Abd El‐Hack et al. 2024).

Spirulina, identified for its remarkable dietary composition, is widely utilized as a dietary ingredient in various food products, enhancing their nutritional value and contributing to overall health, as well as reproductive and productive functions (AlFadhly et al. 2022). Because of its antibacterial, antioxidant, anti‐inflammatory, immune‐boosting and other therapeutic qualities, spirulina is seen as a possible substitute for antibiotics in chicken feed (El‐Shall et al. 2023). Furthermore, via lengthening intestinal villi and boosting the quantity of epithelial cells, especially goblet cells, spirulina powder (SPI) has been demonstrated to boost growth performance, have antiviral qualities and fortify the immune system (Bondar et al. 2023).

This study hypothesizes that dietary incorporation of FAc, HMX and SPI in broiler diets will enhance growth performance and carcass traits through improvements in nutrient utilization, metabolic efficiency and physiological homeostasis, while favourably modulating blood biochemical parameters, antioxidant status and gut microbiota composition, compared with conventional antibiotic supplementation. These variables were selected as integrative indicators of growth, metabolic health, oxidative balance and intestinal function, which collectively reflect the physiological responses of broilers to dietary interventions. Specifically, this research proposes natural feed additives as effective alternatives to antibiotics by enhancing gut health, reducing stress and supporting immune and metabolic functions to maintain productivity. Its originality lies in the comparative evaluation of three additives (FAc, HMX and SPI) versus a traditional antibiotic control, analysing various parameters such as growth performance, carcass characteristics and gut microbiota in a single framework. The study aims to promote sustainable, antibiotic‐free poultry production, addressing concerns over antimicrobial resistance.

## 2 Materials and methods

### 2.1 Herbal Mix (Plant Materials)

The HMX consisted of finely ground powdered forms of *Thymus vulgaris* (thyme), *Pimpinella anisum* (anise), *Capsicum annuum* (chili pepper), *Mentha spicata* (spearmint), *Salvia rosmarinus* (rosemary), *Allium sativum* (garlic) and *Nigella sativa* (black seed). All plant materials were supplied as ready‐to‐use powders and blended in equal proportions. The mixture was purchased as a commercial product from 2 M Group Co., 10th of Ramadan City, Sharqia Governorate, Egypt, and used according to the manufacturer's specifications.

### 2.2 Chemical Characterization

Chemical reagents, including folate, Ciocalteu's phenol reagent (DPPH), 2,2‐diphenyl‐1‐picrylhydrazyl, gallic acid, quercetin, methanol, ethanol and aluminium chloride, were obtained from Merck (Merck KGaA, Darmstadt, Germany).

#### 2.2.1 Preparing the Sample

Approximately 200 mL of 70% methanol were used as the extraction solvent and mixed with 10 g of each finely ground plant material (thyme, anise, chili pepper, spearmint, rosemary, garlic and black seed), as methanol is an efficient solvent for extracting phenolic and flavonoid compounds from plant tissues, thereby ensuring effective recovery of bioactive constituents (Abdel‐Shafi et al. 2019; Ashour et al. 2020). After 3 h of stirring, the liquid was filtered using Whatman No. 2 filter paper. A Buchi water bath B‐480 evaporator operating under vacuum at 45°C was used to extract the methanol from the extract. A Thermoelectron Corporation Heto Power Dry LL 300 Freeze Dryer was then used to lyophilize the extract. Until additional analysis, the resultant dry extract was kept at −20°C (Abd El‐Hack et al. 2018; Ashour et al. 2020).

#### 2.2.2 Total Phenolic Compounds Estimation

The Folin‐Ciocalteu technique was used to measure the total phenolic compounds (TPCs) in the methanolic extracts (1000 mg/mL). Gallic acid (10–1000 mg/mL) was used as the default phenolic ingredient in the creation of a standard curve, with the formula being *y* = 0.001*x* + 0.0563 (*R* ^2^ = 0.9792). One millilitre of each sample or standard, 2 mL of 7.5% sodium carbonate and 3 mL of diluted reagent Folin‐Ciocalteu were combined and incubated for half an hour at 25°C in the dark. A JENWAY 6405 UV/Vis spectrophotometer was used to detect absorbance at 760 nm (Abdel‐Shafi et al. 2019).

#### 2.2.3 Total Flavonoids Estimation

The Ordonez et al. (2006) technique was used to calculate the total flavonoid (TF) content. The quercetin (10–1000 mg/mL) standard curve, *y* = 0.0012*x* + 0.008 (*R* ^2^ = 0.944), was employed. One millilitre of 20 g/L AlCl_3_ ethanol was combined with 1 mL of quercetin extract or solution. The spectrophotometer was used to measure the colour intensity at 420 nm.

#### 2.2.4 Antioxidant Activity Estimation

The antioxidant activity was assessed using the DPPH assay (Ramadan et al. 2008; Osman et al. 2014). Three millilitres of a methanolic DPPH solution were mixed with 1 mL of each sample. After 30‐min incubation, the absorbance was determined at 520 nm utilizing a spectrophotometer. According to Abdel‐Hamid et al. (2017), the extract content that scavenged 50% of the DPPH and ABTS radicals (SC50) was determined.

### 2.3 Experimental Design, Birds and Diets

The Poultry Research Farm, Department of Poultry, Faculty of Agriculture, Zagazig University, Zagazig, Egypt, was the site of the experiment. Every experimental method was carried out in compliance with the Institutional Animal Care and Use Committee (IACUC) guidelines and authorized by the Ethics Committee of the Department of Poultry, Faculty of Agriculture, Zagazig University.

A total of 480 broiler chicks (Ross 308) with comparable initial body weights were divided into eight experimental groups at random in a completely randomized design trial. Ten unsexed chicks made up each of the six replicas in each group. The experiment went on for 35 days. The following were the treatments:

- T1: Negative control (basal diet)
- T2: Positive control (basal diet + 0.5 g Colistin antibiotic/kg diet)
- T3: Basal diet + 2 cm^3^ formic acid/kg diet
- T4: Basal diet + 4 cm^3^ formic acid/kg diet
- T5: Basal diet + 2 g herbal mix/kg diet
- T6: Basal diet + 3 g herbal mix/kg diet
- T7: Basal diet + 0.5 g SPI/kg diet
- T8: Basal diet + 1 g SPI/kg diet

According to NRC (1994), the basal diets were created to satisfy the birds' nutritional needs. According to Table 1, the diets were separated into two phases: starter (1–21 days) and finisher (22–32 days). Birds were kept in wire‐floor cage pens measuring 100 cm × 100 cm × 50 cm, with 10 birds housed per cage, corresponding to a stocking density of 10 birds/m^2^. Chicks were maintained under a 23L:1D lighting program with ad libitum access to feed and water. Artificial lighting was provided for 23 h/day during the first week, followed by a gradual increase in the dark period to 4 h/day for the remainder of the experiment to support normal growth and welfare. During the first week, brooding temperature was maintained at 33°C–35°C, then reduced by 1°C–1.5°C every three days thereafter. All birds were reared under identical environmental, managerial and hygienic conditions throughout the experimental period to minimize confounding influences.

**TABLE 1:** Composition and chemical analysis of basal diets.

Items	Starter (1–21 days)	Finisher (22–32 days)
**Ingredients (%)**		
Yellow corn	54.10	58.7
Soybean meal (44% protein)	34.50	29.5
Corn germ (60% protein)	5.50	5.50
Limestone	1.08	0.95
Dicalcium phosphate	2.00	1.75
Premix^a^	0.30	0.30
Salt	0.30	0.30
L‐lysine	0.29	0.24
DL‐methionine	0.20	0.18
Soybean oil	1.80	2.30
**Calculated analysis** ^b^		
Crude protein (%)	23.12	21.10
Metabolizable energy (Kcal/kg)	3001	3180
Calcium (%)	0.99	0.89
Available phosphorus (%)	0.51	0.46
Potassium (%)	0.54	0.52

^a^ Minerals and vitamins premix manufactured by Multi Vita Animal Nutrition (Tenth of Ramadan City, Sharkia Governorate, Egypt) provides vitamin A 12,000 IU, vitamin D3 2500 IU, vitamin E 20 mg, vitamin K3 2 mg, vitamin B1 2 mg, vitamin B2 5 mg, vitamin B6 2 mg, vitamin B12 0.05 ug, niacin 30 mg, biotin 0.05 ug, folic acid 1 mg, pantothenic acid 10 mg, manganese 60 mg, zinc 50 mg, iron 40 mg, copper 10 mg, iodine 0.6 mg, and selenium 0.3 mg per 1 kg diet. DL‐methionine (manufactured by Evonik Industries, Essen, Germany) and contains 99 % methionine. Lysine = lysine hydrochloride (Evonik Industries) and contains 70% Lysine.

^b^ Calculated according to NRC (1994).

### 2.4 Data Assembly

Weekly, the daily body weight gain (DBWG) and live body weight (LBW) were calculated by weighing each chick separately. In addition, FI and feed conversion ratio (FCR) were computed. Twelve birds from every group were chosen at random to have their carcasses examined at 32 days of age. According to Islamic custom, birds were killed by cutting their jugular veins and letting them bleed for 3–5 min. The weights of the heart, liver, gizzard and carcass were noted upon slaughter.

### 2.5 Blood Biochemical Analysis

After an overnight fast, blood samples were taken in non‐heparinized tubes from the same 12 birds from both groups were then slaughtered. Before biochemical examination, the serum was kept at −20°C after being separated by centrifugation at 5000 rpm for 15 min at 4°C. Commercial diagnostic kits (Bio Diagnostic Co., Giza, Egypt) were used to assess the serum levels of alanine aminotransferase (ALT), aspartate aminotransferase (AST), total protein (TP), albumin, urea and creatinine. In addition, oxidative stress indicators such as reduced glutathione (GSH), glutathione‐S‐transferase (GST), glutathione peroxidase (GPx) and malondialdehyde (MDA) were measured (KGaA, Darmstadt, Germany) (Abd El‐Hack et al. 2017).

### 2.6 Microbiological Examination

Cecum samples (10 g) were extracted from 12 birds per group and homogenized in 90 mL of sterile peptone water. Serial dilutions (10^−1^–10^−7^) of the supernatant were prepared, and total bacterial count (TBC), Lactobacilli, Coliforms, *Escherichia coli*, *Clostridium* and *Salmonella* spp. were quantified using selective media (Oxoid 2006). The bacterial counts were expressed as Log CFU/g.

### 2.7 Statistical Analysis

SAS software was used to analyse the data in a completely random approach utilizing a one‐way ANOVA (SAS Institute Inc. 2001). The linear (L) and quadratic (Q) impacts of rising FAc, HMX and SPI levels were calculated using orthogonal polynomial contrasts. The pen served as the growth performance experimental unit, and the blood and carcass analyses were conducted on individual birds. Duncan's multiple range test was used to find significant differences amongst treatments (*p *< 0.05) (Duncan 1955).

## 3 Results

### 3.1 Live Body Weight and Daily Body Weight Gain

Table 2 presents the influences of dietary supplementation with FAc, HMX and SPI on the LBW and DBWG of birds. Considerable variations (*p *< 0.05) were noted in the overall treatment, linear and quadratic effects for both LBW on Day 32 and DBWG during the periods of Days 22–32 and 1–32. The highest LBW at Day 32 was observed in the spirulina groups, T7 (2034.10 g) and T8 (2007.30 g). Group T2 (2021.25 g), which received the antibiotic Colistin, also showed high LBW. For DBWG during the period from Day 1–32, T7 exhibited the highest rate of weight gain (62.14 g/day), followed closely by T2 (61.74 g/day), indicating consistent weight growth in these groups. The control group (T1) showed the lowest performance. However, no significant variations were observed in LBW on Days 1 and 21 or DBWG from Days 1–21.

**TABLE 2:** Effects of using formic acid (FAc), herbal mix (HMX) or spirulina powder (SPI) on live body weight and daily body weight gain of broiler chickens.

Items	Treatments								SEM	*p* value		
	T1	T2	T3	T4	T5	T6	T7	T8		T	L	Q
**Live body weight (LBW) (g)**												
Day 1	45.24	45.58	45.74	44.57	46.14	45.47	45.69	45.26	0.18	0.690	0.901	0.818
Day 21	970.36	955.31	950.46	975.54	943.18	938.07	987.11	980.76	1.78	0.535	0.532	0.192
Day 32	1935.26^bc^	2021.25^ab^	1890.62^c^	1990.91^ab^	1999.30^ab^	1880.44^c^	2034.10^a^	2007.30^ab^	2.45	0.007	0.005	0.004
**Daily body weight gain (DBWG) (g / day)**												
Days 1–21	44.05	43.32	43.08	44.33	42.72	42.50	44.83	44.55	0.77	0.519	0.534	0.190
Days 22–32	87.72^bc^	96.90^a^	85.47^c^	92.31^ab^	96.01^a^	85.67^c^	95.18^a^	92.32^ab^	1.09	0.001	0.001	0.001
Days 1–32	59.06^bc^	61.74^ab^	57.65^c^	60.82^ab^	61.04^ab^	57.34^c^	62.14^a^	61.31^ab^	1.46	0.007	0.006	0.004

*Note*: Means within the same row with different superscript letters are significantly different (*p *≤0.05). T1: control, T2: 0.5 g Colistin antibiotic/kg diet, T3: 2 cm^3^ FAc, T4: 4 cm^3^ FAc, T5: 2 g HMX, T6: 3 g HMX, T7: 0.5 g SPI, T8: 1 g SPI/kg diet. T, overall impacts of treatments; L, linear impacts of increasing FA, HM and SP levels of broiler; Q, quadratic impacts of increasing FA, HM and SP levels of broiler.

### 3.2 FI and Feed Conversion Ratio

The effects of dietary supplementation with FAc, HMX and SPI on FI, FCR and PI are summarized in Table 3. Except for FCR on Days 1–21, significant linear and quadratic differences were observed between treatments for FI, FCR and PI throughout the study. The herbal mix groups (T5, T6) exhibited the lowest levels of FI. On the other hand, the maximum FI was found in the treatment receiving SPI at 1 g/kg feed (T8). The herbal mix groups (T5, T6) also showed the best FCR values. The control group (T1) had the highest FCR, representing the poorest efficient feed conversion. Regarding the performance index, T5 had the highest PI (138.96), followed by T6 (123.89). Conversely, T1 (110.65) and T3 (105.09) had the lowest PI, indicating suboptimal performance in these groups.

**TABLE 3:** Effects of using formic acid (FAc), herbal mix (HMX) or spirulina powder (SPI) on daily feed intake, feed conversion ratio and performance index (PI) of broiler chickens.

Items	Treatments								SEM	*p* value		
	T1	T2	T3	T4	T5	T6	T7	T8		T	L	Q
**Feed intake (g/day/bird)**												
Days 1–21	58.07^bc^	60.35^ab^	59.26^ab^	60.05^ab^	55.91^c^	58.13^bc^	61.92^a^	62.05^a^	0.52	0.004	0.046	0.017
Days 22–32	149.04^a^	148.27^a^	148.31^a^	150.04^a^	120.63^b^	117.46^b^	147.53^a^	151.62^a^	3.11	0.000	0.060	0.000
Days 1–32	103.56^a^	104.31^a^	103.79^a^	105.05^a^	88.27^b^	87.85^b^	104.23^a^	106.84^a^	1.71	0.000	0.000	0.000
**Feed conversion ratio (g/g)**												
Days 1–21	1.32	1.39	1.38	1.35	1.31	1.37	1.38	1.39	0.11	0.420	0.401	0.539
Days 22–32	1.699^a^	1.530^b^	1.735^a^	1.625^ab^	1.256^c^	1.371^c^	1.539^b^	1.624^ab^	0.14	0.000	0.005	0.000
Days 1–32	1.753^ab^	1.689^ab^	1.800^a^	1.727^ab^	1.446^c^	1.531^c^	1.677^b^	1.742^ab^	0.26	0.000	0.028	0.000
**PI**	110.65^cd^	119.99^bc^	105.09^d^	114.94^abc^	138.96^a^	123.89^b^	121.56^bc^	114.20^abc^	2.27	0.000	0.031	0.009

*Note*: Means within the same row with different superscript letters are significantly different (*p *≤ 0.05). T1: control, T2: 0.5 g Colistin antibiotic/kg diet, T3: 2 cm^3^ FAc, T4: 4 cm^3^ FAc, T5: 2 g HMX, T6: 3 g HMX, T7: 0.5 g SPI, T8: 1 g SPI/kg diet. T, overall impacts of treatments; L, linear impacts of increasing FA, HM and SP levels of broiler; Q, quadratic impacts of increasing FA, HM and SP levels of broiler.

### 3.3 Carcass Characteristics

Table 4 shows the impacts of dietary supplements with FAc, HMX and SPI on carcass traits at the end of the experiment. No significant variations were identified in the majority of carcass metrics throughout the treatments compared to the control group. Nevertheless, notable differences were detected in gizzard weight, with T5 (2 g HMX) showing the highest gizzard weight relative to other treatments. Furthermore, the control group exhibited the highest spleen weight compared to all other treatments.

**TABLE 4:** Effects of using formic acid (FAc), herbal mix (HMX) or spirulina powder (SPI) on carcass characteristics of broiler chicken.

Items	Treatments								SEM	*p* value		
	T1	T2	T3	T4	T5	T6	T7	T8		T	L	Q
Pre‐slaughtered weight	2187.00	2282.00	2281.00	2042.00	2120.00	2090.00	2081.00	2119.00	4.02	0.922	0.334	0.779
Carcass (g)	1582.00	1651.00	1665.00	1517.00	1548.00	1538.00	1522.00	1533.50	2.98	0.960	0.367	0.944
Breast meat (g)	971.00	1018.00	1054.00	969.00	948.00	968.00	960.00	936.00	2.84	0.908	0.323	0.721
Drum meat (g)	613.00	636.00	607.00	548.00	604.00	559.00	562.00	591.00	2.90	0.898	0.416	0.495
Liver (g)	48.90	56.50	55.30	49.80	45.60	51.00	45.00	53.00	1.70	0.191	0.103	0.660
Heart (g)	11.30	10.20	8.90	9.50	8.60	10.40	9.10	10.10	0.34	0.415	0.845	0.826
Gizzard (g)	25.40^b^	23.60^b^	28.20^b^	23.70^b^	32.00^a^	23.40^b^	25.60^b^	25.90^b^	0.85	0.031	0.020	0.018
Spleen (g)	3.30^a^	2.20^b^	2.60^ab^	2.30^b^	2.20^b^	2.10^b^	2.10^b^	2.20^b^	0.98	0.050	0.011	0.081

*Note*: Means within the same row with different superscript letters are significantly different (*p *≤ 0.05). T1: control, T2: 0.5 g Colistin antibiotic/kg diet, T3: 2 cm^3^ FAc, T4: 4 cm^3^ FAc, T5: 2 g HMX, T6: 3 g HMX, T7: 0.5 g SPI, T8: 1 g SPI/kg diet. T, overall impacts of treatments; L, linear impacts of increasing FA, HM and SP levels of broiler; Q, quadratic impacts of increasing FA, HM and SP levels of broiler.

### 3.4 Blood Biochemical Parameters

The effects of FAc, HMX and SPI supplementation on liver and kidney function are presented in Table 5. Linear and quadratic differences (*p *< 0.05) were detected for TP, ALB, GLOB, ALT, AST, urea and creatinine levels among the treatments. The herbal mix groups (T5 and T6) showed the highest levels of TP, ALB and GLOB compared to the other treatments, while the SPI diet at 1 g/kg (T8) showed the lowest values. In addition, the herbal mix groups (T5 and T6) had the highest AST levels and the lowest ALT values, suggesting a favourable liver enzyme profile. The lowest urea and creatinine levels were found in T4 (4 cm^3^ FAc) and T3 (2 cm^3^ FAc), respectively. In contrast, the maximum urea and creatinine levels were noted in T5 (2 g HMX) and T6 (3 g HMX), respectively.

**TABLE 5:** Effects of using formic acid (FAc), herbal mix (HMX) or spirulina powder (SPI) on liver and kidney function of broiler chickens.

Items	Treatments								SEM	*p* value		
	T1	T2	T3	T4	T5	T6	T7	T8		T	L	Q
TP (g/dL)	4.37^b^	4.13^c^	2.65^e^	3.29^d^	6.41^a^	6.36^a^	2.79^e^	2.44^f^	0.31	0.000	0.000	0.000
ALB (g/dL)	2.33^b^	2.27^b^	1.28^d^	1.57^c^	3.10^a^	3.34^a^	1.48^c^	1.28^d^	0.15	0.000	0.000	0.000
GLOB (g/dL)	2.04^b^	1.86^b^	1.37^c^	1.72^b^	3.31^a^	3.02^a^	1.31^c^	1.16^c^	0.16	0.000	0.000	0.000
A/G (%)	1.14	1.22	0.93	0.91	0.94	1.11	1.13	1.10	0.04	0.248	0.619	0.193
ALT (IU/L)	53.37^a^	51.87^a^	27.40^b^	19.37^d^	10.49^f^	10.36^f^	15.37^e^	23.50^c^	1.36	0.000	0.000	0.000
AST (IU/L)	106.03^b^	118.47^a^	44.62^d^	55.03^c^	121.58^a^	119.30^a^	34.73^e^	43.77^d^	1.65	0.000	0.000	0.000
Urea (mg/dL)	12.53^a^	12.20^a^	8.10^bc^	5.57^c^	12.22^a^	9.03^b^	7.47^bc^	8.63^b^	0.54	0.000	0.001	0.046
Creatinine (mg/dL)	0.56^a^	0.51^b^	0.34^d^	0.41^c^	0.42^c^	0.54^a^	0.37^d^	0.36^d^	0.16	0.000	0.000	0.000

*Note*: Means within the same row with different superscript letters are significantly different (*p *≤ 0.05).

T1: control, T2: 0.5 g Colistin antibiotic/kg diet, T3: 2 cm^3^ FAc, T4: 4 cm^3^ FAc, T5: 2 g HMX, T6: 3 g HMX, T7: 0.5 g SPI, T8: 1 g SPI/kg diet. TP: total protein, ALB: albumin, GLOB: globulin, AST: aspartate aminotransferase, ALT: alanine aminotransferase. T, overall impacts of treatments; L, linear impacts of increasing FA, HM and SP levels of broiler; Q, quadratic impacts of increasing FA, HM and SP levels of broiler.

### 3.5 Antioxidant Parameters

Dietary supplementation with FAc, HMX and SPI improved the antioxidant status of the broiler chickens (Table 6). MDA concentrations were lower in all treatment groups in comparison with the control group, with the smallest levels detected in T5 (2 g HMX). The activities of GSH, GST and GPx were highest in T5 (2 g HMX), indicating a significant improvement in antioxidant defence.

**TABLE 6:** Effects of using formic acid (FAc), herbal mix (HMX) or spirulina powder (SPI) on blood antioxidant enzymes of broiler chickens.

Items	Treatments								SEM	*p* value		
	T1	T2	T3	T4	T5	T6	T7	T8		T	L	Q
GPx (U/mg protein)	150.50^d^	167.36^b^	162.00^cd^	159.00^cd^	179.34^a^	158.19^cd^	166.00^bc^	179.60^a^	1.25	0.000	0.000	0.000
GSTs (U/mg protein)	4.54^cd^	5.63^bc^	6.46^ab^	4.07^d^	7.24^a^	6.72^ab^	5.79^bc^	6.15^ab^	0.23	0.001	0.012	0.001
GSH (U/mg protein)	62.31^d^	71.53^c^	74.43^c^	62.65^d^	90.89^a^	71.65^c^	80.32^b^	79.48^b^	1.91	0.000	0.000	0.005
MDA (nmol/mL)	6.35^a^	4.81^b^	3.72^bc^	4.58^bc^	3.41^c^	3.56^bc^	3.54^bc^	3.57^c^	0.20	0.001	0.000	0.023

*Note*: Means within the same row with different superscript letters are significantly different (*p *≤0.05). T1: control, T2: 0.5 g Colistin antibiotic/kg diet, T3: 2 cm^3^ FAc, T4: 4 cm^3^ FAc, T5: 2 g HMX, T6: 3 g HMX, T7: 0.5 g SPI, T8: 1 g SPI/kg diet. T, overall impacts of treatments; L, linear impacts of increasing FA, HM and SP levels of broiler; Q, quadratic impacts of increasing FA, HM and SP levels of broiler.

Abbreviations: GPx: glutathione peroxidase, GSTs: glutathione‐S‐transferase, GSH: glutathione reduced, MDA: malondialdehyde.

### 3.6 Microbial Analysis of the Broiler Cecum

Table 7 presents the caecal microbial count in response to different FAc, HMX and SPI treatments. Significant differences (*p *< 0.05) were found in the TBC. The highest TBC values were recorded in the T5 (2 g HMX) and T6 (3 g HMX) groups. The group supplemented with 2 cm^3^ of formic acid (T3) exhibited the highest levels of *Lactobacilli*, indicating beneficial effects on gut health. Notably, the control group (T1) had the maximum counts of *Salmonella* and *E. coli* compared to all treatment groups. The lowest levels of these harmful bacteria were found in T3, T4 and T5 treatments (2 cm^3^ FAc, 4 cm^3^ FAc and 2 g HMX). However, no significant effects were observed on *Clostridium* or *Coliform* counts across the treatments.

**TABLE 7:** Effects of using formic acid (FAc), herbal mix (HMX) or spirulina powder (SPI) on caecal microbial count (Log CFU/g) of broiler chickens.

Items	Treatments								SEM	*p* value		
	T1	T2	T3	T4	T5	T6	T7	T8		T	L	Q
TBC	8.47^a^	8.28^b^	8.05^d^	8.01^e^	8.23^a^	8.19^a^	8.16^b^	8.07^c^	0.02	0.000	0.000	0.000
*Lactobacilli*	7.37^b^	7.12^c^	8.12^a^	7.08^d^	7.44^b^	7.42^b^	7.14^c^	7.17^c^	0.05	0.000	0.000	0.000
*Coliform*	6.41	6.09	6.02	6.05	6.34	6.37	6.07	6.14	0.07	0.790	0.916	0.696
*E. coli*	5.37^a^	5.10^b^	4.04^e^	4.00^e^	4.21^c^	4.27^c^	4.09^d^	4.25^c^	0.09	0.000	0.000	0.000
*Salmonella*	5.27^a^	4.08^c^	3.17^d^	3.08^d^	3.09^d^	4.29^bc^	4.38^bc^	4.59^b^	0.16	0.000	0.000	0.000
*Clostridium*	4.74	4.16	4.38	4.39	4.11	4.13	4.42	4.38	0.07	0.478	0.612	0.108

*Note*: Means within the same row with different superscript letters are significantly different (*p *≤ 0.05). T1: control, T2: 0.5 g Colistin antibiotic/kg diet, T3: 2 cm^3^ FAc, T4: 4 cm^3^ FAc, T5: 2 g HMX, T6: 3 g HMX, T7: 0.5 g SPI, T8: 1 g SPI / kg diet. T, overall impacts of treatments; L, linear impacts of increasing FA, HM and SP levels of broiler; Q, quadratic impacts of increasing FA, HM and SP levels of broiler.

Abbreviations: TBC: total bacterial count.

## 4 Discussion

The findings from this study demonstrate that the treated birds exhibited superior growth performance in comparison with the control group. Specifically, on Day 32, LBW of birds in the SPI treatments, T7 and T8, were the highest. These outcomes are consistent with the results of Alwaleed et al. (2021), who reported that Cobb broilers complemented with 10 g/kg of SPI exhibited significant body weight gain and improved FCR over 35 days. Likewise, Fathi et al. (2018) found that supplementation with 0.7 and 0.9 g of SPI/kg of diet improved growth rates, blood issues and serum biochemistry in broilers. In addition, Qureshi et al. (1996) observed enhanced body weights in White Cornell Leghorns and broiler chicks when fed diets with varying levels (10, 100, 1000 and 10,000 ppm) of *Spirulina platensis*, further corroborating the positive effects of spirulina on broiler growth.

Spirulina's rich nutritional content is responsible for its growth‐promoting properties, which include high‐quality protein, essential vitamins, minerals and antioxidants. Spirulina contains approximately 60%–70% protein, which is of excellent quality with a well‐balanced amino acid profile. This makes it an excellent source of protein for broilers, supporting muscle development and weight gain (Lestingi et al. 2024). In addition, spirulina is abundant in vital minerals like potassium, magnesium and iron, vitamins such as B‐complex and vitamin E, and fatty acids like gamma‐linolenic acid (GLA), all of which promote metabolic efficiency and overall growth (Marjanović et al. 2024). The polysaccharides and phycocyanin in spirulina further enhance immune function, improving resistance to disease and fostering an environment conducive to optimal growth (Selim et al. 2025).

Moreover, the addition of SPI in the feed may stimulate enlarged FI and improve nutrient absorption, which could directly contribute to better weight gain (Salah et al. 2025). Spirulina's ability to enhance feed conversion efficiency by promoting beneficial gut microbiota, improving nutrient digestibility and stimulating metabolic activity has been well‐documented (Alghamdi et al. 2024; Prates 2025).

The utilization of HMX in this study demonstrated notable improvements in FCR and PI, while reducing FI across all age groups and experimental periods, suggesting enhanced growth efficiency. These results align partially with Adli et al. (2024), who reported that herbal supplementation in poultry diets can improve FCR and reduce mortality, although no significant effects were observed on average daily gain (ADG) or FI. Additional studies also support these findings; for instance, Lipiński et al. (2019), Jin et al. (2020) and El‐Abasy et al. (2025) observed that herbs with bioactive compounds can positively influence growth performance and feed efficiency. The presence of bioactive compounds such as allicin in garlic, thymol in thyme and anethole in anise can regulate energy metabolism, promote protein anabolism and stimulate immune functions by reducing oxidative stress and enhancing antioxidant status (Urban et al. 2024; Deeb et al. 2025). Similarly, Mohammed et al. (2018) and Deeb et al. (2024) reported that herbs like garlic and black seed can reduce FI, likely due to their strong flavour and bioactive compounds with antioxidant properties.

Regarding gut microbiota and digestive physiology, herbs such as thyme, pepper and anise contain essential oils and phytochemicals that stimulate gizzard activity, enhance feed grinding and promote the secretion of digestive enzymes, supporting nutrient digestibility (Phillips et al. 2023; Rehman et al. 2025). Increased gizzard activity, stimulated by these herbal compounds, may lead to muscular hypertrophy and improved nutrient absorption, thereby enhancing growth performance. This is consistent with Liu et al. (2023), who reported that herbal supplements can enhance gizzard function and digestive efficiency. Furthermore, black seed and chili pepper have been shown to activate gizzard musculature, improve feed breakdown and increase nutrient extraction, further contributing to growth promotion and feed efficiency (Vázquez‐González et al. 2023; Negm et al. 2025).

Moreover, to promote growth, HMX supplementation positively affected plasma protein levels, with the highest levels of TP, ALB and GLOB observed in the HMX groups. This may be credited to the improvement of gut health and digestive enzyme action, which improves nutrient absorption and facilitates protein synthesis. Herbs such as black seed, *M. spicata* (spearmint) and chili pepper are rich in bioactive compounds like flavonoids, alkaloids and essential oils, which have been shown to stimulate the production of albumin and globulin in the liver (Faheem et al. 2022). Furthermore, compounds like capsaicin may improve lipid metabolism, indirectly promoting protein synthesis (Wang et al. 2022). Anethole from anise plays a key role in modulating hormonal pathways, particularly those related to protein metabolism, ensuring efficient synthesis of plasma proteins like albumin and globulin (Nasir and Yabalak 2024).

A considerable conclusion of this experiment is the reduced levels of ALT in birds supplemented with the HMX. This indicates a protective effect on liver health, as elevated ALT levels are often a marker of liver damage. Herbs such as *S. rosmarinus* (rosemary) and *A. sativum* (garlic) contain strong antioxidants like rosmarinic acid and organo‐sulphur compounds, which shield liver cells from oxidative stress and lipid peroxidation, thus reducing ALT leakage (Emerald 2024). These results are supported by studies showing that antioxidants from herbal sources, including capsaicin, carvacrol and thymol, help maintain liver health by neutralizing reactive oxygen species (ROS) (Surai et al. 2024).

In addition, SPI supplementation, particularly at 1 g/kg, resulted in significantly lower AST levels, indicating improved hepatic integrity and reduced hepatocellular stress. Spirulina is reported to enhance liver antioxidant defence by upregulating enzymes such as superoxide dismutase (SOD) and GPx, which limit oxidative damage to hepatocytes and support normal liver metabolic and detoxification pathways (AlThobaiti 2023; Roueintan et al. 2025). This antioxidant‐mediated protection helps maintain liver function and prevents enzyme leakage into circulation, and reduces toxic metabolites such as ammonia, lipid peroxidation products and bacterial endotoxins (Eilam et al. 2023). Spirulina's high levels of phycocyanin, beta‐carotene and GLA protect hepatocytes from damage, thereby reducing AST leakage into the bloodstream (Nongalleima et al. 2019). Furthermore, spirulina's anti‐inflammatory properties, through the suppression of pro‐inflammatory cytokines like TNF‐α and IL‐6, contribute to a reduction in AST leakage, as inflammation can exacerbate tissue damage and enzyme release (Toughan et al. 2018).

The FAc groups also demonstrated lower levels of creatinine and urea, which is in line with the results of Abd El‐Hack et al. (2024), who noted that FAc supplement in broilers led to a reduction in these metabolites. By decreasing the pH in the gut, increasing the efficiency of digestive enzymes, and promoting nitrogen utilization, all of which reduce urea production, FAc facilitates the digestion of proteins and the absorption of amino acids (Fabay et al. 2022). FAc also works by inhibiting harmful gut bacteria that produce ammonia, which further lowers urea levels (Hossain et al. 2024). Furthermore, FAc reduces muscle breakdown, as evidenced by lower creatinine levels, and helps maintain kidney and liver health by reducing oxidative stress and inflammation (Eklund et al. 2005; Ungureanu et al. 2024).

The HMX groups exhibited significantly higher antioxidant enzyme activities, including GPx, GST and GSH, alongside lower concentrations of MDA, which is an indicator of lipid peroxidation. Polyphenols, flavonoids and essential oils found in herbs like thyme, rosemary and black seed promote the synthesis and activity of endogenous antioxidants, such as GPx and GST, which detoxify harmful substances and protect cells from oxidative damage (Marino et al. 2022). Also, the antioxidant enzymes GPx and GST work synergistically with GSH, which plays a crucial role in detoxifying ROS and guarding cells from oxidative harm. The increase in GSH levels observed in this study likely contributed to the enhanced antioxidant defence system and reduced oxidative damage (Arif et al. 2023). The synergistic interactions between the bioactive compounds in herbs strengthen the overall antioxidant capacity, providing enhanced protection against oxidative stress (Rajčević et al. 2022).

Spirulina supplementation, particularly at 1 g/kg, also exhibited significant antioxidant effects. The abundance of phycocyanin, beta‐carotene, tocopherols and other phenolic compounds found in spirulina. The high levels of phycocyanin, beta‐carotene, tocopherols and other phenolic compounds found in spirulina encourage the activity of antioxidant enzymes such as GPx, which lowers DNA damage, peroxidation of lipid and oxidative stress (Abdelkhalek et al. 2015). Phycocyanin, in particular, directly boosts GPx activity, while also reducing inflammation, which is a known contributor to oxidative stress (Mohiti et al. 2021).

Finally, the experiment demonstrated that FAc supplementation (T3, 2 cm^3^ of formic acid) resulted in the highest populations of *Lactobacilli* and the lowest levels of TBC, *E. coli* and *Salmonella*, indicating improved gut microbial balance. This aligns with previous research showing that OAs reduce gastrointestinal pH, inhibit the growth of pathogenic bacteria and support the proliferation of beneficial microflora, thereby enhancing gut health (Attia et al. 2018; Nair et al. 2018; Gerzilov and Hristakieva 2025). Short‐chain OAs such as formic, acetic and butyric acids exert direct antimicrobial effects against pathogenic bacteria like *Salmonella* and *E. coli*, improving intestinal integrity and decreasing the need for antibiotics (Regassa and Nyachoti 2018; Ricke et al. 2020; Abd El‐Hack et al. 2024). Moreover, OAs have been shown to stimulate the activity of beneficial digestive enzymes in the ileum and increase the ratio of commensal microbes in the cecum, further supporting nutrient digestibility and gut health (Palamidi and Mountzouris 2018; Khorasgani et al. 2025).

Specific OAs, including citric, fumaric and lactic acids, acidify the stomach, creating an unfavourable environment for pH‐sensitive pathogens, while formic, acetic, butyric, propionic and sorbic acids can directly disrupt Gram‐negative bacterial cell walls, reducing their populations in the gut (Dittoe et al. 2018; Negm et al. 2023). These combined influences of pH reduction and direct antimicrobial action contribute to improved gut morphology, increased villus height, enhanced nutrient absorption and overall better growth performance in broilers, corroborating multiple studies on the efficacy of OAs as antibiotic alternatives (Fathima et al. 2022; Li et al. 2023).

## 5 Conclusion

The present study indicated that different concentrations of FAc, HMX and SPI increased LBW and DBWG, especially in the SPI groups. Moreover, HM groups (2 g HMX) enhanced blood antioxidant enzyme levels, FCR, PI, TP, ALB, GLOB and ALT levels. Furthermore, FAc groups improved gut bacteria levels and renal function. These treatments demonstrated potential as innovative additions to broiler diets, offering consumers a wholesome organic product. Future research should focus on optimizing dosage concentrations of FAc, HMX and SPI to maximize growth, health benefits and cost‐effectiveness. Long‐term studies are needed to evaluate their effects on meat quality, including flavour, tenderness, and nutritional content, to understand their impact on the final product offered to consumers. Investigating the sustainability and economic feasibility of using FAc, HMX, and SPI in poultry diets is also essential, along with their potential to replace antibiotics and improve gut health. Given the impact on gut microbiota and antioxidant enzymes, further research into their immune‐modulating effects could enhance the understanding of their role in promoting gut health in poultry. In conclusion, while the study provides promising results regarding the use of FAc, HMX and SPI in broiler diets, additional research is needed to investigate their long‐term influences, optimize supplementation levels and assess their broader applications in sustainable poultry farming.

## Author Contributions

**Elwy A. Ashour**: conceptualization, investigation, writing – original draft, software. **Saad A. Al‐Ardhi**: validation, writing – review and editing, formal analysis, data curation. **Dalia A. A. Elsayed**: visualization, writing – review and editing, resources, writing – original draft. **Mahmoud Moustafa**: funding acquisition, writing – original draft, writing – review and editing, visualization, formal analysis, data curation. **Islam M. Youssef**: investigation, validation, visualization, data curation, software. **Mohammad M.H. Khan**: data curation, validation, visualization, writing – original draft, writing – review and editing. **Mohamed E. Abd El‐Hack**: conceptualization, investigation, methodology, data curation, writing – review and editing, writing – original draft.

## Funding

This work was funded by the Deanship of Research and Graduate Studies at King Khalid University through the Large Research Project under grant number RGP2/113/47.

## Ethics Statement

This experiment was performed at the Poultry Farm, Department of Poultry, Faculty of Agriculture, Zagazig University, Egypt. All procedures followed the regional animal care protocols (IACUC).

## Conflicts of Interest

The authors declare no conflicts of interest.

## Data Availability

Data will be available upon reasonable request.
